# Enhanced antiamoebic efficacy of PHMB via methyltransferase inhibition to maintain drug sensitivity and block encystation

**DOI:** 10.1186/s13071-026-07597-6

**Published:** 2026-08-04

**Authors:** Jui-Chi Wu, Chun-Hsien Chen, Jian-Ming Huang

**Affiliations:** 1 Department of Medical Science, College of Life Sciences and Medicine, National Tsing Hua University, Hsinchu, Taiwan; 2 Institute of Molecular and Cellular Biology, College of Life Sciences and Medicine, National Tsing Hua University, Hsinchu, Taiwan; 3 Department of Parasitology, College of Medicine, Chang Gung University, Taoyuan, Taiwan; 4 School of Medicine, College of Life Sciences and Medicine, National Tsing Hua University, Hsinchu, Taiwan

**Keywords:** *Acanthamoeba*, Methyltransferase, Polyhexamethylene biguanide, 5-Azacytidine

## Abstract

**Background:**

Acanthamoeba keratitis (AK) is a severe ocular infection. The primary treatment is polyhexamethylene biguanide (PHMB), but its efficacy is limited due to cyst resistance. This study investigated the role of DNA methylation in *Acanthamoeba* encystation and the potential efficacy of combining 5-azacytidine (5-AzaC; a methyltransferase inhibitor) with PHMB treatment.

**Methods:**

Encystation was induced in *Acanthamoeba castellanii* using encystation buffer, followed by calcofluor white staining. Gene expression levels of virulence genes and the methyltransferase gene were assessed by quantitative PCR. The cell viability assay of *A. castellanii* on Statens Seruminstitut Rabbit Cornea (SIRC) cells was evaluated to determine functional virulence. Overexpression of methyltransferase via electroporation was established to further investigate its function.

**Results:**

qPCR results revealed upregulated methyltransferase expression during encystation. Calcofluor white staining showed significantly reduced cyst formation in *A. castellanii* treated with 5-AzaC. This treatment also induced significant transcriptional reduction in virulence genes. Consistently, 5-AzaC-treated *A. castellanii* did not exhibit increased cytotoxicity against SIRC cells. Overexpression of the methyltransferase increases the encystation ratio. Combination treatment with PHMB and 5-AzaC significantly decreased *A. castellanii* survival.

**Conclusions:**

Inhibiting DNA methylation enhances the antiamoebic effects of PHMB, offering a potential AK treatment strategy. DNA methylation may serve as a critical regulatory switch in *A. castellanii*, governing both morphological transformation and virulence genes expression. This work expands our understanding of epigenetic regulation in protozoa.

**Graphical Abstract:**

**Figure:**
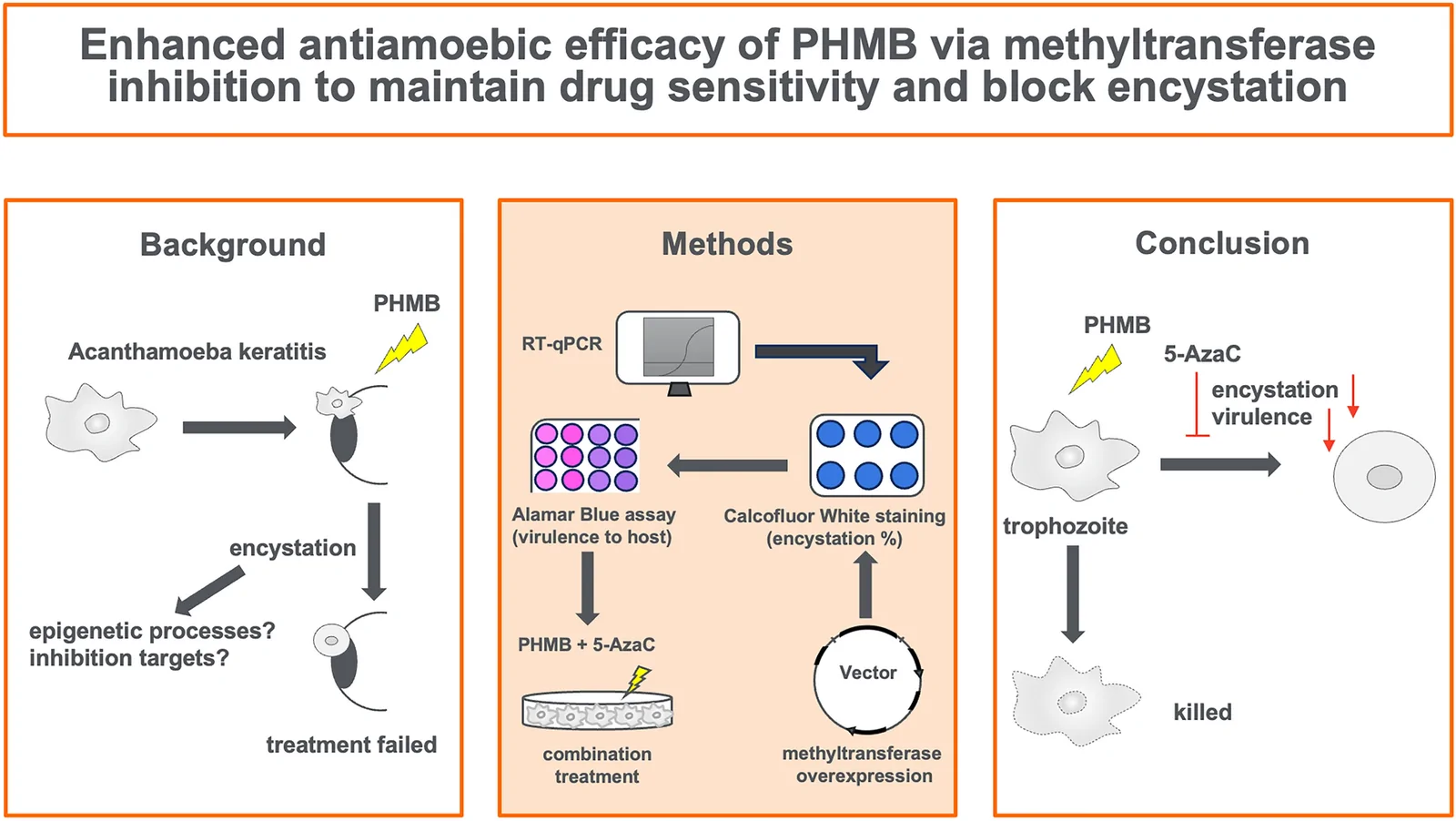

## Background

*Acanthamoeba* (family: Acanthamoebidae) is a non-flagellated, free-living protozoan that moves at approximately 0.8 μm/s [1]. It is widely found in natural environments, including swamps, lakes, rivers and mud, as well as in swimming pools [2].

*Acanthamoeba* is a single-celled non-photosynthesizing organism. Its life cycle includes two forms: trophozoite and cyst. The trophozoite (approximately 25–40 µm) contains major organelles, such as the nucleus and mitochondria. The genome is approximately 45.1 Mb, including 7193 genes and 32,836 expression subunits [3]. In natural environments, *Acanthamoeba* primarily obtains nutrients by engulfing organisms, such as bacteria, yeast and algae. It replicates by binary fission in the trophozoite form, with a generation time of 8–24 h [4]. When *Acanthamoeba* encounters low nutrient levels and unfavorable conditions, it undergoes encystation, transforming from a trophozoite into a double-walled cyst (approximately 13–23 µm), which demonstrates enhanced resistance to external factors [5]. The outer wall of the cyst comprises proteins and polysaccharides and the inner wall is mainly composed of cellulose [6]. In favorable environments, *Acanthamoeba* excysts to form trophozoites, resuming binary fission for replication.

*Acanthamoeba* is an opportunistic pathogen that causes infections in humans, leading to serious diseases, particularly in immunocompromised patients or contact lens wearers [7]. There are three main routes of infection: (1) central nervous system invasion through the nasal cavity and other components of the respiratory tract, leading to granulomatous amoebic encephalitis [8]; (2) invasion through skin wounds, causing disseminated infection [9]; and (3) invasion though areas of corneal damage (the most common route), leading to Acanthamoeba keratitis (AK). The first case of AK was reported in the UK in 1974. Subsequently, the rapid increase in the number of contact lens wearers has led to a rise in AK cases. Because repeated wearing and removing of contact lenses can damage the cornea, wearing improperly cleaned soft contact lenses provides an easy route for *Acanthamoeba* invasion [2, 10, 11 ].

AK is an infectious disease that poses a serious threat to vision and may even require the removal of the eyeball in severe infections. Patients with AK often present with significant eye pain and symptoms including photophobia, ring-like stromal infiltrates, corneal epithelium defects and eyelid swelling [12]. Currently, the primary treatment for AK is 0.08% polyhexamethylene biguanide (PHMB) monotherapy [13], but some patients respond poorly due to the high levels of drug resistance exhibited by the cyst form. This is attributed to the polysaccharide structure of the cyst wall, which influences physiological and drug resistance, making it difficult for existing biochemical agents to penetrate and subsequently eradicate mature cysts [14].

There are three main categories of epigenetic modifications: DNA methylation, histone modification and nucleosome positioning [15]. DNA methylation is crucial for regulating several biological processes, including chromatin structure, gene silencing, genomic imprinting and X-chromosome inactivation [16–18]. DNA methyltransferase (DNMT) 5, a cytosine DNMT biochemically characterized in the Fungi and Protista kingdoms, is a maintenance enzyme responsible for CpG methylation in the human pathogen *Cryptococcus neoformans*. Because DNMT5 is essential for complete virulence, it is a potential target for antifungal drugs [19–22]. A functional DNMT in *Plasmodium falciparum* (PfDNMT) plays a role in regulating virulence gene expression and transcription elongation [23]. A DNMT in *Entamoeba histolytica* (Ehmeth) was shown to be inhibited by 5-azacytidine (5-AzaC), significantly reducing the parasite’s cytotoxicity toward mammalian cells and its capacity to induce liver abscesses in hamsters [24]. The mechanisms by which DNA methylation influences gene expression in *Acanthamoeba* remain unclear. However, a comparative microarray study demonstrated the upregulation of 701 genes in *Acanthamoeba* during encystation [25], suggesting significant differences in mRNA expression levels before and after encystation. Therefore, we speculate that inhibiting specific genes through DNA methylation may inhibit *Acanthamoeba* encystation. 5-AzaC is a DNMT inhibitor that restores tumor suppressor gene expression by inducing DNA demethylation and is widely used for treating myelodysplastic syndrome and various cancers [26]. Furthermore, it was reported that 5-AzaC-induced DNMT inhibition reduces the percentage of *Acanthamoeba* encystation [27]. This work aimed to identify target gene involved in the inhibition of encystation and to explore potential inhibitory compound.

## Methods

### *Acanthamoeba castellanii* culture

*Acanthamoeba castellanii* Neff strain was obtained from ATCC (cell line 30010; Manassas, VA) and cultured axenically at 28 ºC in cell culture flasks containing peptone yeast extract glucose broth (20 g/L proteose peptone, 2 g/L yeast extract, 0.1 M glucose, 4 mM MgSO_4_, 3.4 mM sodium citrate, 0.9 mM Fe(NH_4_)_2_(SO_4_)_2_, 1.3 mM Na_2_HPO_4_ and 2 mM K_2_HPO_4_ [pH 6.5]) [28].

### SIRC culture

The Statens Seruminstitut Rabbit Cornea (SIRC) cell line was cultured in complete Dulbecco’s Modified Eagle Medium (DMEM) supplemented with 10% (v/v) heat-inactivated fetal bovine serum (FBS; Gibco, USA) and 1% (v/v) penicillin–streptomycin (Simply, Taiwan). The culture was maintained at 37 °C in a humidified atmosphere containing 5% CO_2_.

### Total RNA isolation and cDNA synthesis

Total RNA was extracted using a Direct-zol RNA Miniprep Kit (Cat. No. R2052; Zymo Research, USA). The concentration and A_260 nm_/A_280 nm_ ratio of mRNA were measured using a NanoDrop One spectrophotometer (Thermo Fisher Scientific, USA). A Moloney Murine Leukaemia Virus Reverse Transcription Kit (Protech, Taiwan) was used for cDNA synthesis in a final reaction volume of 20 μL under the following incubation conditions: 25 °C for 10 min, 42 °C for 60 min and 70 °C for 10 min.

### Quantitative PCR (qPCR)

qPCR was performed using GoTaq qPCR Master Mix (Promega, USA) according to the manufacturer’s protocol to evaluate the expression levels of virulence genes and the methyltransferase gene (*ACA1_267260*). The primer pairs targeted *Acanthamoeba* actin-related protein 2 (*ARP2*; forward: 5′-GCT GTC TTG ACC CTC TAC GC-3′, reverse: 5′-AGC GAG AAG CCC TCG TAC AC-3′; target amplicon size: 101 bp) [29], methyltransferase (*ACA1_267260*; forward: 5′-TGC AAG ATC ACC TCG CTC-3′, reverse: 5′-ACA TCA CAC AAT CGA AGC-3′; target amplicon size: 132 bp), cysteine protease 3 (*CP 3*; forward: 5′-CGA TGC CTC GCA CAA CTC CTT C-3′, reverse: 5′-CCA CGA GTT CTT GAC GAG CCA-3′; target amplicon size: 148 bp), serine endopeptidase (*SEP*; forward: 5′-GAA GGC GCT CAC CGA ATA CAT-3′, reverse: 5′-GGT TCG TCA TCT GCT GAT AGC C-3′; target amplicon size: 104 bp), mannose binding protein (*MBP*; forward: 5′-AGG GCG AGA CCT ACG ATA GC-3′, reverse: 5′-CCT CGT AGA CGA AGG TGA GG-3′; target amplicon size: 165 bp) [30], and *Acanthamoeba* laminin binding protein (*AhLBP*; forward: 5′-CCA ACA CCG ACT CTC CTC TC-3′, reverse: 5′-CTC CTC AGG GTC ACG GTA GA-3′; target amplicon size: 183 bp) [30]. qPCR reactions were performed in a final reaction volume of 20 μL on a CFX96 Real-Time PCR Detection System (Bio-Rad, USA). Amplification was carried out using a preset program (3-step SYBR Amp + Melt). The melting curve was analyzed to confirm amplification specificity. Relative gene expression levels were calculated using the 2^−ΔΔCt^ method.

### *Acanthamoeba castellanii* encystation

*Acanthamoeba* *castellanii* trophozoites were collected and washed twice with Page modified Neff amoeba saline (PAS; 120 mg NaCl, 4 mg MgSO_4_ × 7H_2_O, 3 mg mM CaCl_2_, 142 mg Na_2_HPO_4_ and 136 mg KH_2_PO_4_), followed by culture at a density of 5 × 10^6^ cells per T-25 flask in 4 mL of encystation buffer (EB; 95 mM NaCl, 5 mM KCl, 8 mM MgSO_4_, 0.4 mM CaCl_2_, 1 mM NaHCO_3_ and 20 mM Tris–HCl [pH 9.0]) for 24 h [31].

### Percentage of *A. castellanii* encystation

*Acanthamoeba* *castellanii* trophozoites were cultured at a density of 2.5 × 10^6^/mL in a six-well plate. For the inhibition assay, cells were cultured in PAS, EB, or EB supplemented with 10 μM 5-AzaC for 24 h [27, 32 ]. For the overexpression assay, transfected *A. castellanii* and control cells were incubated with EB. Cysts were stained with 1% calcofluor white stain (Sigma, USA) and counted under a ZOE Fluorescent Cell Imager (Bio-Rad) to determine the percentage of encystation.

### Cell viability assay

SIRC cells were seeded in 96-well plates at a density of 5 × 10^4^ cells/well and cultured at 37 °C in 5% CO₂ for 48 h. After incubation, the culture medium was removed, and the cells were washed once with phosphate-buffered saline (PBS). For the negative control group (representing 100% cell viability), SIRC cells received 90 μL of serum-free DMEM without amoebae for 24 h. For the infection groups, SIRC cells were co-cultured with *A. castellanii* pretreated with EB (serving as the positive control for amoeba-mediated cytotoxicity) or EB supplemented with 10 μM 5-AzaC for 24 h. For both infected groups, infections were performed at multiplicities of infection (MOIs) of 1, corresponding to 1 × 10^5^ cells/well. This pathogenic co-culture model was adapted from a previously described protocol with minor modifications [33].

Following 24 h of co-culture, 10 μL of AlamarBlue reagent (G-Biosciences, USA) was added to each well and incubated for 1 h. Fluorescence intensity was measured using a microplate reader (BMG Labtech, Germany) at excitation and emission wavelengths of 540 and 590 nm, respectively. Cell viability was expressed as a percentage relative to the negative control group.

### Cloning of methyltransferase

The *ACA1_267260* coding sequence was synthesized commercially (JetFa Biotech Co., Ltd) with an NdeI site preceding the initiation codon and a SpeI site after the codon. The synthesized sequence was provided in the pET-21a(+) vector for initial propagation. The insert was then excised from pET-21a(+) using NdeI and SpeI, purified, and ligated into the pGAPDH-EGFP vector. [34] All plasmids were transformed to DH5α competent *E. coli* for replication and construction.

### Cell electroporation of *A*. *castellanii*

For electroporation, cells were counted using a hemocytometer and centrifuged at 2500 rpm for 5 min to remove the medium.* Acanthamoeba castellanii* cells were resuspended in PYG to a final count of 5 × 10^6^ cells/mL and placed in an Eppendorf tube. Ten micrograms of plasmid DNA were added to the Eppendorf tube, followed by PYG to a final volume of 800 μL. The mixture was gently mixed and dispensed into a 4—mm cuvette. Using Gene Pulser Xcell^™^ (Bio-Rad, USA), the protocol was set as follows: 600 V, 25 μF. After electroporation, the cuvettes containing cells were placed on ice for 10 min, and cells were transferred to a T-75 flask containing PYG for incubation at 28 °C overnight. Stable transformants were selected using 10 μg/mL Geneticin (G418).

### PCR verification of *A. castellanii* stable transfectants

To confirm the successful integration of the expression vectors, genomic DNA was extracted from stable transfectants using the Gene-Spin Genomic DNA Isolation Kit (Protech, Taiwan) according to the manufacturer’s instructions. PCR amplification was performed using Fast-Run^™^ 2X Taq Master Mix (Protech, Taiwan).

For the identification of the pGAPDH-EGFP-methyltransferase construct, a methyltransferase-specific forward primer (methyltransferase _forward: 5′-TGC AAG ATC ACC TCG CTC-3′) and a vector-specific reverse primer (pGAPDH-EGFP_reverse:5′-CCG GAC ACG CTG AAC TTG − 3′) were utilized. This primer pair was designed to yield a 654-bp product, comprising the methyltransferase fragment (553 bp) and a portion of the vector backbone (101 bp). For the empty vector control, a pair of pGAPDH-EGFP-specific primers (forward: 5′-TGT GCT GGT GGT CTC GGT3′, reverse: 5′-CCG GAC ACG CTG AAC TTG − 3′) was used to amplify a 210-bp fragment. The PCR products were analyzed by electrophoresis on a 1% agarose gel and visualized to ensure the presence of bands of the expected sizes.

### Percentage of *A. castellanii* survival following 5-AzaC treatment

*Acanthamoeba castellanii* was cultured at a density of 5 × 10^5^ cells/mL in a 12-well plate. The cells were incubated with EB or a combination of EB and 5-AzaC for 96 h to induce cyst formation. Subsequently, they were treated with 0.01% PHMB. Dead cells were identified using trypan blue staining. The percentage of surviving cysts was quantified by counting under light microscopy.

### Statistical analysis

Data were expressed as the mean ± standard deviation (SD) from three independent experiments. Statistical significance was analyzed using Student *t*-test. P-values < 0.05 were considered statistically significant.

## Results

### Relationship between methyltransferase and encystation in *A. castellanii*

**Fig.1:**
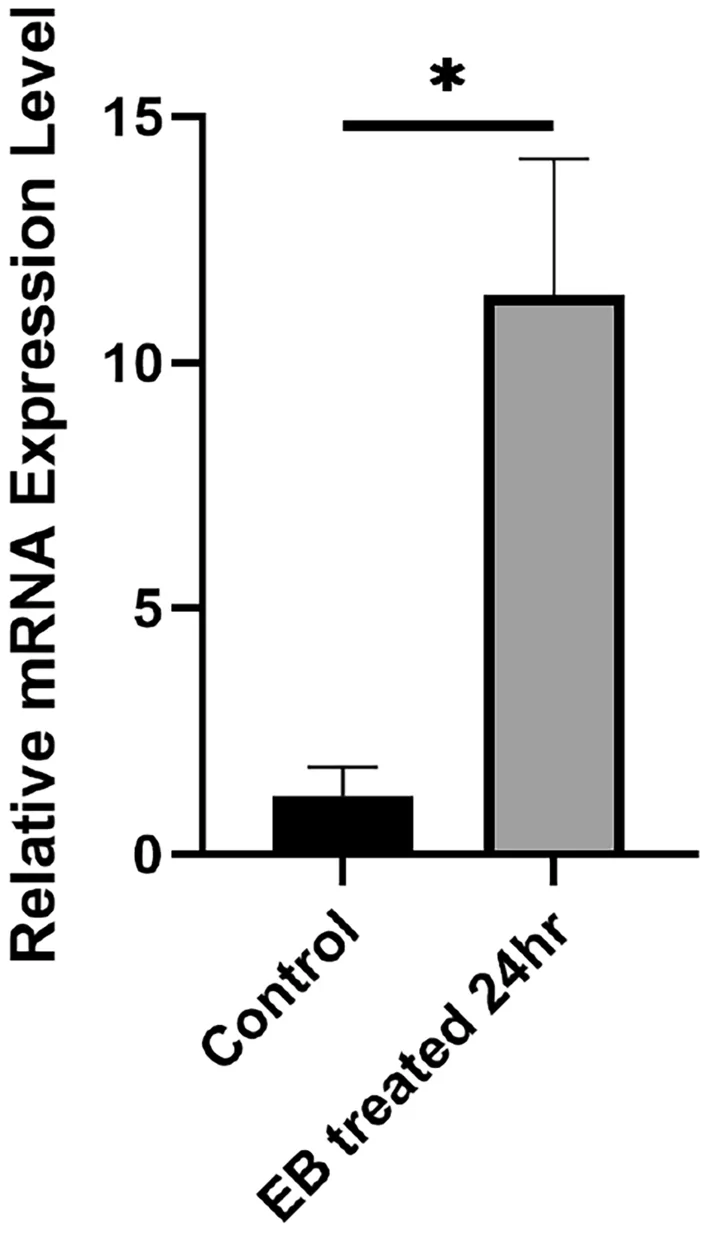
mRNA expression levels of methyltransferase in *A. castellanii*. *A. castellanii* was cultured in EB for 24 h. The mRNA expression level of methyltransferase was calculated using the 2^−ΔΔCt^ method. The expression level of *ARP2* was used as an internal control. Bar graphs present the mean ± SD (*P ≤ 0.05)

Methylation of DNA cytosine bases reduces the accessibility of regulatory elements to transcription factors via multiple mechanisms, often acting synergistically with histone deacetylation to regulate gene transcription [17]. Given that mRNA expression levels differ significantly before and after *Acanthamoeba* encystation [25], we hypothesized that DNA methylation was involved in this process. Accordingly, we used qPCR to assess the expression of the *A. castellanii* methyltransferase gene under encystation-inducing conditions. Methyltransferase expression was upregulated 9.6-fold after 24 h in EB (Fig. 1).

### Effect of the methyltransferase inhibitor 5-AzaC on *A. castellanii* encystation

**Fig. 2:**
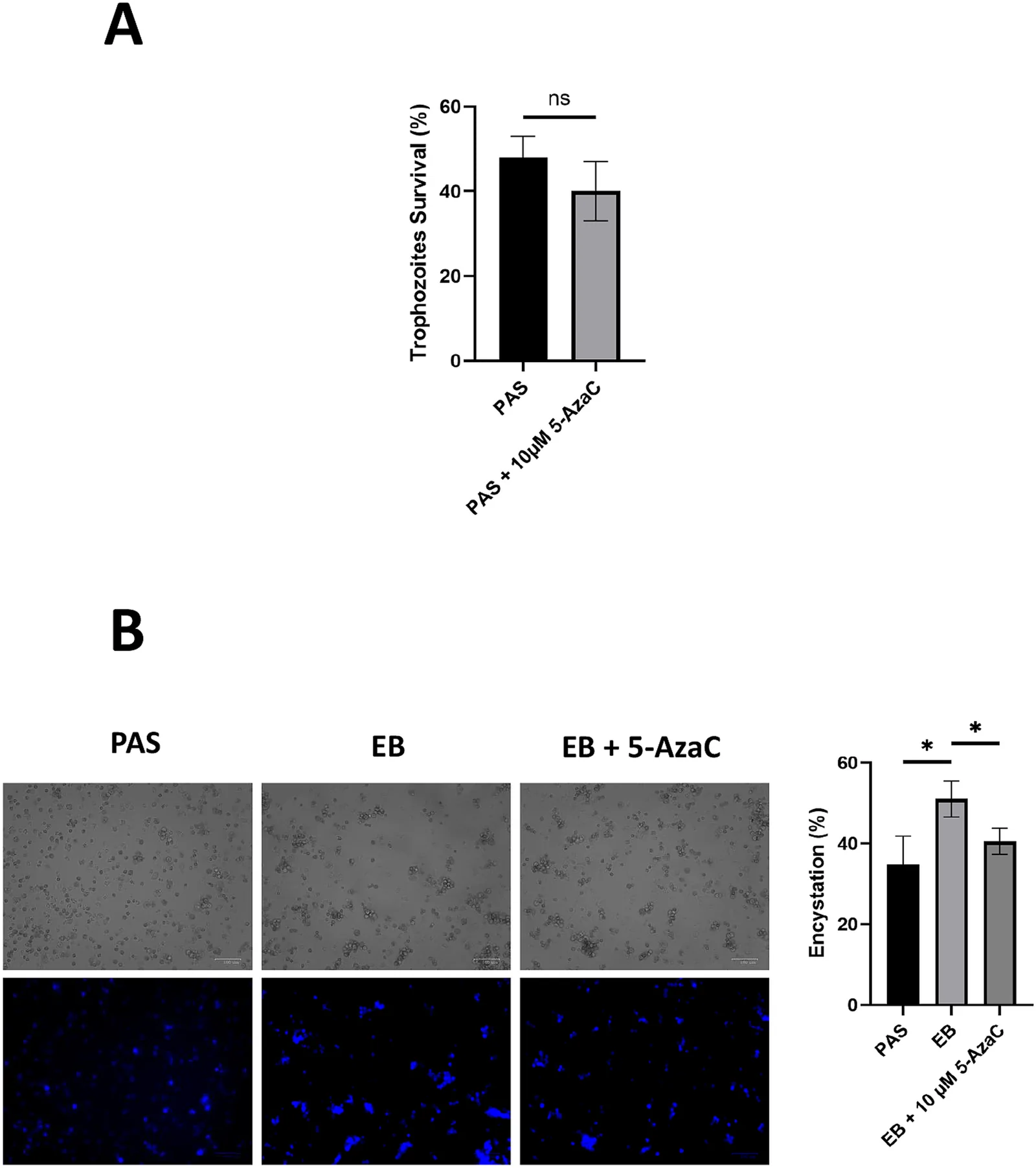
Survival and percentage of encystation of *A. castellanii* treated with methyltransferase inhibitor. **A** Percentage of surviving *A. castellanii* treated with PAS or PAS + 5-AzaC for 24 h. **B** Left panel: fluorescent microscopy of *A. castellanii* stained with 1% calcofluor white following incubation with PAS, EB, or EB+5-AzaC for 24 h. Right panel: percentage of encystation of *A. castellanii* treated with PAS, EB, or EB + 5-AzaC for 24 h. Bar graphs present the mean ± SD (*P ≤ 0.05)

5-AzaC is one of the most extensively studied DNMT inhibitors. It contains modified cytidine residues [35] that are incorporated into DNA during replication, thereby inhibiting DNMT activity [26]. To evaluate the effect of DNA methylation inhibition on *A. castellanii* encystation, we first investigated whether 5-AzaC influenced trophozoite viability. No significant difference was observed between the treatment and control groups (Fig. 2A). Next, we assessed the percentage of encystation following 24 h of EB treatment. The percentage of encystation was 51% in the control group. In contrast, the percentage of cells undergoing encystation following 5-AzaC treatment was significantly reduced to 41% (Fig. 2B).

### Effect of the methyltransferase inhibitor 5-AzaC on *A. castellanii* virulence

**Fig.3:**
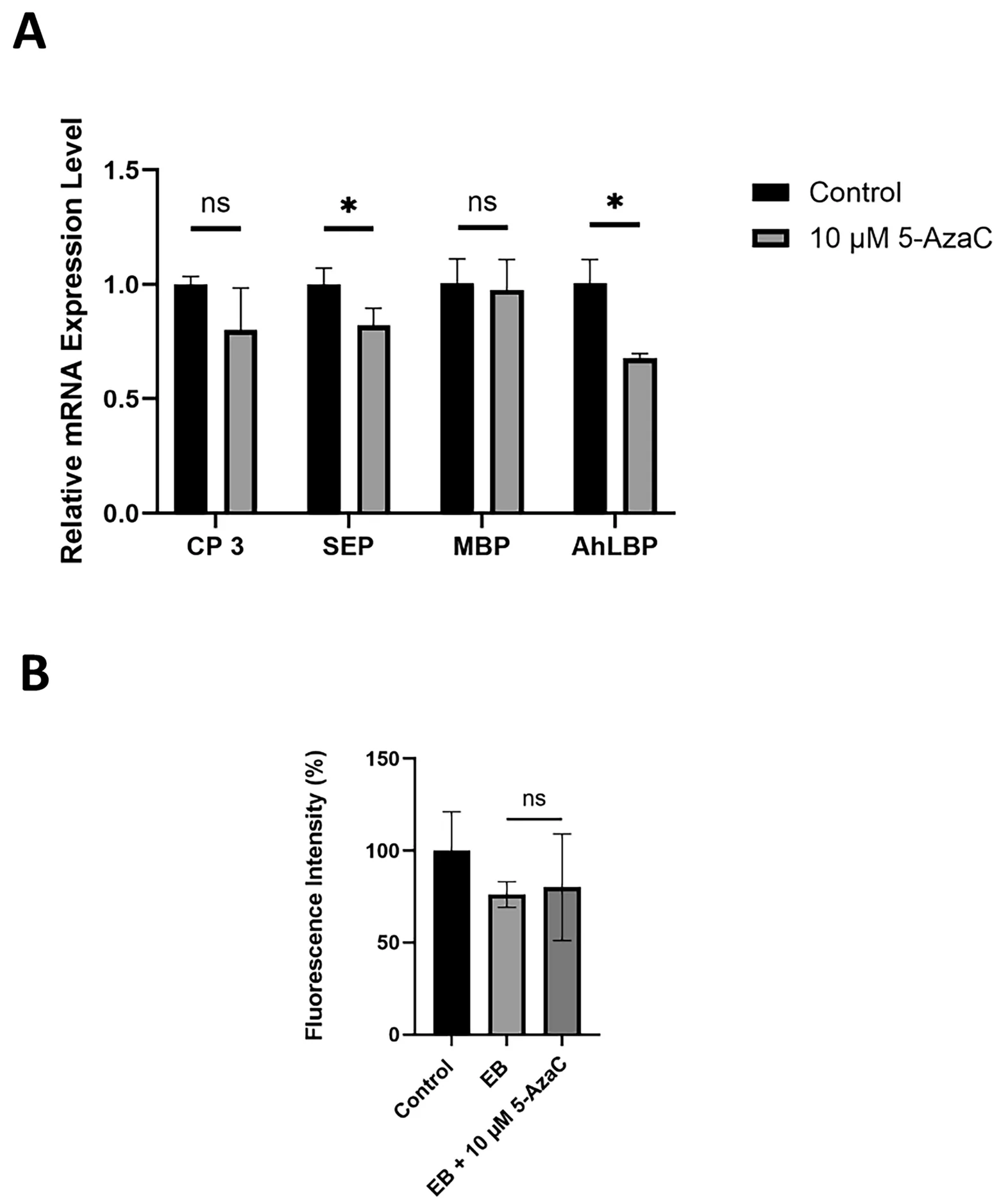
Virulence of *A. castellanii* treated with methyltransferase inhibitor. *A. castellanii* was cultured in EB or EB + 5-AzaC for 24 h. **A** The mRNA expression levels of *CP 3*, *SEP*, *MBP* and *AhLBP* genes were determined using the 2^−ΔΔCt^ method. The expression levels of *ARP2* were used as an internal control. **B** SIRC cells viability following co-culture with *A. castellanii* treated with EB, or EB + 5-AzaC was quantified using the Alamar Blue assay. Bar graphs present the mean ± SD (*P ≤ 0.05)

DNMTs are essential for the virulence of diverse pathogens, ranging from fungi to parasitic protists. Thus, we investigated the effect of the 5-AzaC on *A. castellanii* virulence. qPCR was performed to assess the expression levels of virulence-associated genes, including *CP 3*, *SEP*, *MBP*, and *AhLBP*. Among these, *SEP* and *AhLBP* were significantly downregulated following EB+5-AzaC treatment, reaching 0.82-fold and 0.68-fold (Fig. 3A).

To further evaluate the functional consequences, cell viability assays were conducted to determine the impact of 5-AzaC-treated *A. castellanii* on SIRC cells. No significant difference in cell viability was observed between the two infection groups, indicating that 5-AzaC does not increase the cytotoxicity of *A. castellanii* (Fig. 3B). Importantly, although inhibition of DNA methyltransferase reduced the encystation ratio, potentially increasing the proportion of trophozoites, this did not lead to enhanced virulence.

### The function of methyltransferase in *A. castellanii*

**Fig. 4:**
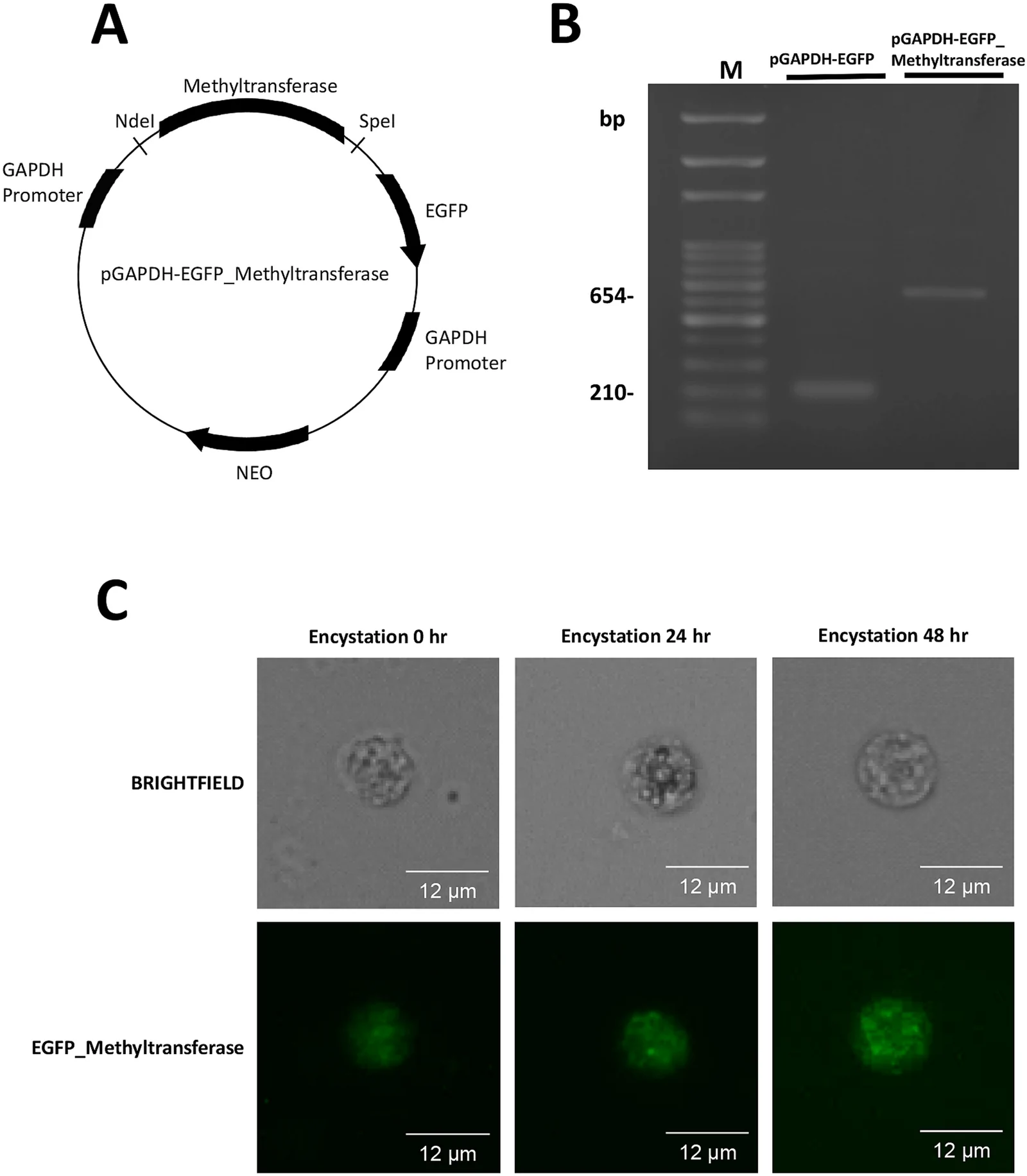
Methyltransferase overexpression in *A. castellanii*. **A** Schematic of the pGAPDH-EGFP-methyltransferase vector. **B** Genomic DNA of *A. castellanii* transfected in the pGAPDH-EGFP-methyltransferase vector detected by PCR. **C** *A. castellanii* transfected with pGAPDH-EGFP and pGAPDH-EGFP-methyltransferase vector (green) incubated for 48 h and examined using a fluorescence microscope

To determine whether methyltransferase of *A. castellanii* can affect encystation, the enzyme was overexpressed by the pGAPDH-EGFP vector. A methyltransferase fragment was inserted into the pGAPDH-EGFP vector using NdeI/SpeI sites (Fig. 4A). After transfection in *A. castellanii* by electroporation for 14 days, the pGAPDH-EGFP-methyltransferase vector was expressed. To confirm successful transfection of the vector into *A. castellanii*, DNA was extracted and amplified using a forward methyltransferase primer and a reverse pGAPDH-EGFP primer. For the control group the empty pGAPDH-EGFP vector was similarly verified using vector-specific primers. (Fig. 4B). The EGFP-methyltransferase fusion protein was also visualized in *A. castellanii* using a ZOE™ Fluorescent Cell Imager (Bio-Rad, USA) for 48 h (Fig. 4C).

**Fig. 5:**
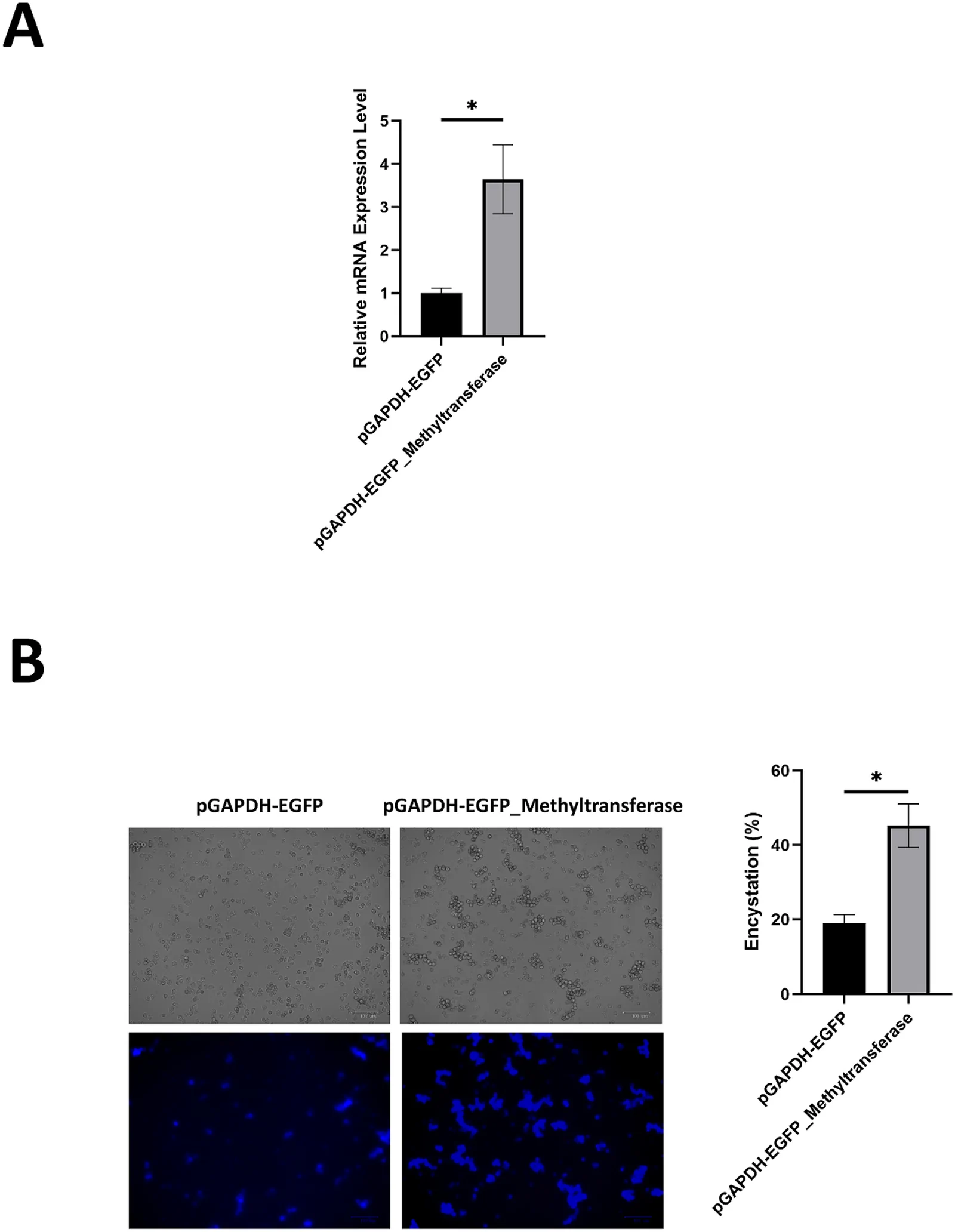
Methyltransferase overexpression promotes *A. castellanii* encystation. **A** mRNA expression levels of methyltransferase were measured via qPCR in transfected cells following 24 h of EB treatment. The mRNA expression level was calculated using the 2^−ΔΔCt^ method. The expression level of *ARP2* was used as an internal control. **B** Percentage of encystation was assessed after 24 h of EB treatment. Bar graphs present the mean ± SD (*P ≤ 0.05)

Following the successful establishment of stable transfection, methyltransferase mRNA expression levels were quantified via qPCR. The results revealed a 3.6-fold increase compared to the control group (Fig. 5A). Finally, the encystation percentage was assessed; while the control group showed an encystation percentage of 19%, the transfected *A. castellanii* exhibited a marked increase to 45% (Fig. 5B). Collectively, these findings demonstrate that methyltransferase plays a critical role in promoting *A. castellanii* encystation.

### Effect of a combination drug treatment on *A. castellanii*

**Fig.6:**
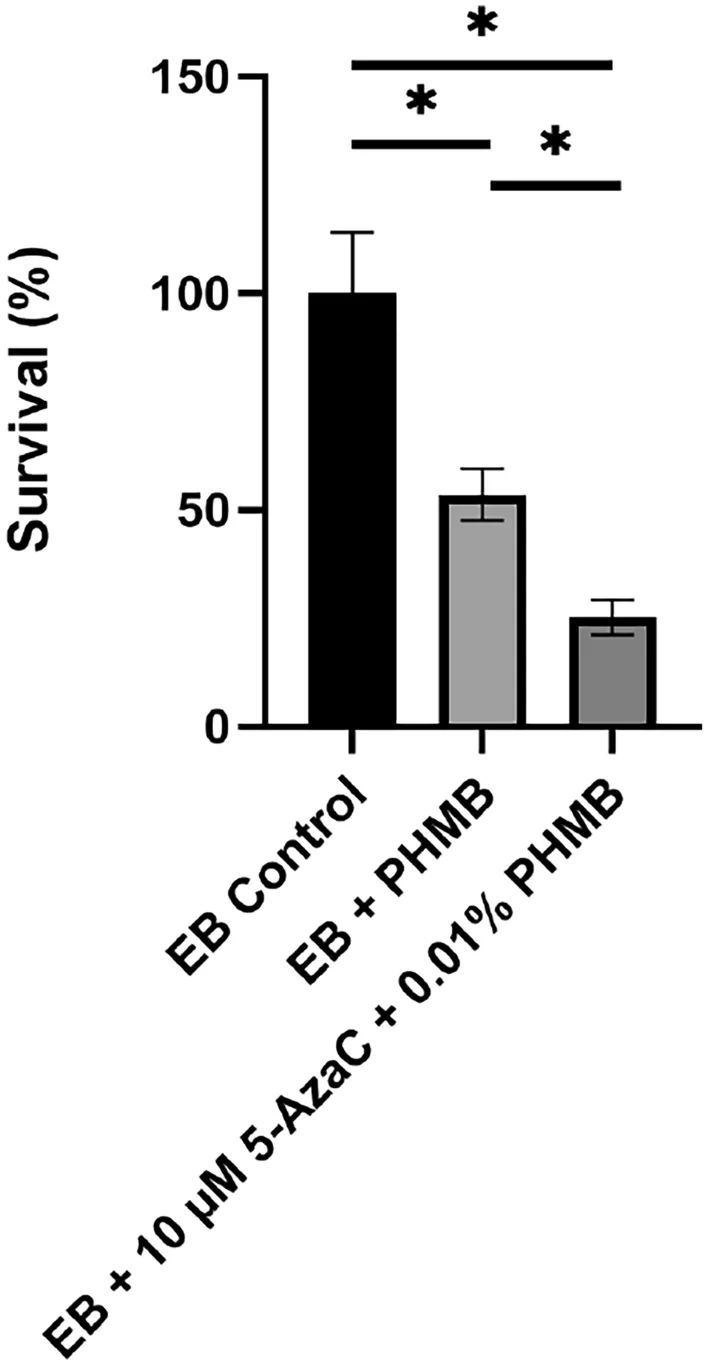
Combination effect of 5-AzaC and PHMB on *A. castellanii*. *A. castellanii* was incubated with EB or EB + 5-AzaC for 96 h to ensure that almost all had formed cysts. Bar one represents the control group, while bars two and three represent treatment with PHMB, after 96 h of encystation. Both groups were treated with 0.01% PHMB for 1 h. The percentage of surviving cysts was calculated by counting the remaining cysts of *A. castellanii* under light microscopy. Bar graphs present the mean ± SD (*P ≤ 0.05)

Because 5-AzaC reduced the percentage of encystation, we next investigated whether inhibiting DNA methylation enhanced the amoebicidal activity of PHMB by treating *A. castellanii* cysts with 0.01% PHMB alone or in combination with 5-AzaC. The percentage of surviving cysts in the EB control group was 54%. In contrast, the percentage of surviving cysts decreased to 25% when PHMB was combined with 5-AzaC, representing an approximately 2-fold reduction (Fig. 6). These observations suggest that 5-AzaC suppresses encystation in *A. castellanii*, thereby enhancing the amoebicidal effect of PHMB.

## Discussion

In this study, we found that the expression of the methyltransferase domain-containing protein in the *Acanthamoeba castellanii* Neff strain (GenBank accession number *ACA1_267260*) was significantly upregulated following EB treatment, suggesting its potential role in regulating encystation-related genes. Furthermore, inhibition of this gene using 5-AzaC reduced the percentage of encystation. These findings suggest that inhibition of DNA methylation suppresses *A. castellanii* encystation. While the single-treatment reduction in the initial encystation rate appeared modest (from 51 to 41%), the combination of 5-AzaC and 0.01% PHMB enhanced amoebicidal activity substantially, with the cyst survival rate decreasing from 54 to 25%. It is probable that although 5-AzaC only partially decreases the overall encystation ratio, it effectively prevents a critical proportion of cells from transforming into fully mature, highly resistant cysts, thereby maintaining them in a drug-sensitive state. However, further ultrastructural analyses using TEM or SEM and prolonged encystation assays will be required to support this hypothesis. In addition to impeding morphological transformation, targeting this epigenetic pathway altered the virulence profile of *A. castellanii*. Pharmacological inhibition by 5-AzaC induced significant transcriptional downregulation of key virulence genes. Crucially, the cell viability assays using SIRC cells demonstrated that 5-AzaC treatment of *A. castellanii* did not enhance its pathogenicity toward host cells. This indicates that methyltransferase inhibition does not trigger compensatory mechanisms that enhance trophozoite virulence. To validate the functional role of methyltransferase, we established a stable overexpressing transfectant, which exhibited a significant increase in the encystation ratio. These findings demonstrate that methyltransferase plays a critical role in promoting *A. castellanii* encystation.

Beyond our observation, other epigenetic mechanisms in *Acanthamoeba* further support the concept that coordinated transcriptional reprogramming drives encystation. Class II histone deacetylases (HDACs), which generally promote transcriptional repression through chromatin condensation, are required for cyst development, as pharmacological inhibition significantly reduces *A. castellanii* encystation [36]. Similarly, AcPRMT5, a protein arginine methyltransferase associated with transcriptional regulation, is upregulated during late encystation, and its silencing impairs mature cyst formation [37]. These findings are consistent with the increased methyltransferase expression observed in our study. Conversely, AcPRMT1, which is commonly associated with transcriptional activation, is also induced during encystation, and its knockdown prevents mature cyst formation [38]. Together, these findings indicate that encystation requires dynamic epigenetic remodeling involving both transcriptional repression and activation, rather than a single regulatory pathway. While DNA methylation and repressive factors may suppress trophozoite-associated programs, activating mechanisms may contribute to the expression of encystation-related genes required for differentiation and survival. To further elucidate the underlying molecular mechanisms, future studies should evaluate the transcriptional profiles of key encystation-related genes under 5-AzaC treatment. Additionally, utilizing whole-genome bisulfite sequencing will allow us to identify specific gene loci that undergo DNA methylation during encystation, and to verify whether 5-AzaC treatment successfully reduces methylation at these targets.

Our previous research demonstrated that inhibition of P-type ATPase by ouabain significantly reduced the percentage of *Acanthamoeba* encystation and combination treatment with PHMB enhanced antiamoebic activity [39]. Similarly, this study demonstrated that methyltransferase inhibition also decreased the percentage of encystation and enhanced the therapeutic effect of PHMB without increasing cytotoxicity of *Acanthamoeba* to host cells. Moreover, the role of methyltransferase was confirmed through overexpression, which directly correlated its function with the encystation process. These findings suggest that combination therapy targeting multiple pathways is a promising strategy for treating AK. Thus, further investigation is warranted.

DNA methylation levels are nearly undetectable in newborn larvae of the parasitic nematode *Trichinella spiralis*. However, they surge dramatically in muscle tissue and during transformation into adults, showing a mosaic pattern dominated by CpG methylation. This increase correlates with differential methylation of parasitism-related genes, such as DNase II, where hypermethylated promoters repress expression in non-relevant stages, facilitating stage-specific adaptations and morphological transitions, such as muscle invasion and cyst formation [40]. Velázquez-Gómez et al. reported that inhibition of protein arginine methyltransferase 5 reduced trophozoite viability and inhibited encystation in the pathogenic amoeba *E. invadens*,[41] establishing methylation regulation as a key mechanism governing protozoan differentiation. Similarly, DNA methylation may also influence morphology in other protozoans, including *Acanthamoeba* encystation, where environmental stressors trigger transformation. Our study confirmed the role of methylation in *Acanthamoeba*, demonstrating that methyltransferase inhibitors, such as 5-AzaC, reduce the percentage of encystation. Epigenetic mechanisms further modulate encystation, suggesting conserved pathways across parasites.

Various combination therapies against *Acanthamoeba* infection have been investigated. Conventional treatment typically relies on combination treatment using biguanides with diamidines, which mainly act by enhancing amoebicidal and cysticidal effects [42]. Later studies have developed new combination strategies. For instance, Polat et al. demonstrated that a combination treatment of miltefosine and PHMB achieved the highest antiamoebic activity by enhancing the amoebicidal effect against both trophozoites and cysts in a rat keratitis model [43]. Similarly, Bagga et al. reported that the early addition of Rose Bengal photodynamic antimicrobial therapy to PHMB + chlorhexidine treatment effectively resolved most AK cases and improved visual outcomes [44]. Although these studies demonstrated promising results, they mainly focused on additive effects to enhance cysticidal effects. Thus, such treatment may fail in strains with highly drug-resistant cysts. In contrast, our study aimed to inhibit the encystation process itself, thereby preventing cyst formation and enhancing the effectiveness of antiamoebic agents.

Regarding the clinical implementation of this pharmacological strategy, we envision a combination topical therapy (e.g., combination eye drops) containing both 5-AzaC and conventional biguanides like PHMB. Mechanistically, 5-AzaC acts as a chemosensitizing agent to suppress encystation and downregulate pathogenic factors. Simultaneously, PHMB can effectively exert its amoebicidal activity on these trophozoites. This approach should be administered during the early stages of infection to prevent the formation of drug-resistant chronic reservoirs. Further evaluation across a wider range of clinical isolates, alongside in vivo studies utilizing animal keratitis models, is warranted to confirm the broad-spectrum efficacy of this strategy and to optimize the synergistic concentrations and dosing regimens of this dual-action formulation.

## Conclusions

This in vitro study demonstrates that DNA methylation-associated regulation may represent a potential therapeutic target for AK. We found that methyltransferase expression is significantly upregulated during encystation, and pharmacologically inhibiting the enzyme with 5-AzaC effectively impedes cyst formation. Importantly, 5-AzaC treatment also downregulates virulence genes without increasing cytotoxicity to host cells, confirming a favorable safety profile. The essential role of methyltransferase in driving this transformation process was further validated through overexpression assays, which resulted in a marked increase in the encystation ratio. These findings suggest that epigenetically regulated transformation pathways may be conserved across parasites. Finally, combining 5-AzaC with PHMB yielded superior antiamoebic efficacy compared to PHMB monotherapy. By blocking the transformation to highly resistant mature cysts, this epigenetic inhibition strategy may overcome a major hurdle in current AK management and warrants further in vivo investigation in animal models.

## Data Availability

Data supporting the main conclusions of this study are included in the manuscript.
